# Clinical evidence of initiating a very low dose of sacubitril/valsartan: a prospective observational analysis

**DOI:** 10.1038/s41598-021-95787-w

**Published:** 2021-08-11

**Authors:** Hyoeun Kim, Jaewon Oh, Sanghyup Lee, Jaehyung Ha, Minjae Yoon, Kyeong-hyeon Chun, Chan Joo Lee, Sungha Park, Sang-Hak Lee, Seok-Min Kang

**Affiliations:** 1 Cardiology Division, Department of Internal Medicine, Severance Cardiovascular Hospital, Cardiovascular Research Institute, Yonsei University College of Medicine, 50-1, Yonsei-Ro, Seodaemun-gu, Seoul, 03722 Korea; 2 Department of Health Promotion, Health Promotion Center, Severance Hospital, Yonsei University Health System, Seoul, Korea

**Keywords:** Drug discovery, Cardiology

## Abstract

Sacubitril/valsartan is superior to enalapril in reducing the risks of cardiovascular death and preventing hospitalization in patients with heart failure and reduced ejection fraction (HFrEF). However, patients often do not receive sacubitril/valsartan because of concerns about hypotension. We examined the feasibility of initiating sacubitril/valsartan at a very low dose (VLD) in potentially intolerant patients with HFrEF and subsequent dose up-titration, treatment persistence and outcomes. We analyzed 206 patients with HFrEF grouped according to starting sacubitril/valsartan dose. The VLD group (n = 106) commenced 25 mg twice daily, and the standard-dose (SD) group (n = 100) started on ≥ 50 mg twice daily. Baseline systolic blood pressure was 103 ± 12 mmHg vs. 119 ± 14 mmHg in the SD group (P < 0.001). The maximal target dose achievement rate was higher in the SD group (27.0% vs 9.4%, p = 0.001) and the VLD group experienced more dose up-titrations and fewer down-titrations than the SD group. The VLD group had a decrease in N-terminal prohormone of brain natriuretic peptide (NT-proBNP) similar to the SD group and a similar increase in left ventricular ejection fraction. There were no significant differences in symptomatic hypotension, worsening renal function, hyperkalemia, cardiovascular mortality, and rehospitalization due to HF between the two groups during follow-up period. In patients considered by the treating physician likely to be intolerant of sacubitril/valsartan, initiation with 25 mg twice daily was generally possible and patients remained in therapy, with similar decreases in NT-proBNP and increases in left ventricular ejection fraction to those observed in patients receiving SD sacubitril/valsartan.

## Introduction

In the PARADIGM-HF [Prospective Comparison of Angiotensin Receptor/Neprilysin Inhibitor (ARNI) Sacubitril/Valsartan (SV) with Angiotensin-converting Enzyme Inhibitor (ACEi) to Determine Impact on Global Mortality and Morbidity in Heart Failure (HF) trial], SV (LCZ696) significantly reduced cardiovascular (CV) mortality and HF hospitalization, compared to enalapril in patients with HF with reduced ejection fraction (HFrEF)^1^. Based on this result, current guidelines give a Class I recommendation for the use of SV in patients with HFrEF^2,3^. Despite the current guideline recommendations, the prescription rate of SV remains still low in “real-world” clinical practice^4–8^. In PARADIGM-HF, the pre-SV treatment dose of enalapril during run-in was 10 mg twice daily. However, patients in clinical practice may not be tolerant of this relatively high dose of an ACEi or equivalent dose of angiotensin receptor blocker (ARB). Recent studies showed that sub-optimal ACEi or ARB dosing may be one of the main reason for non-use of SV^9,10^. Moreover, when SV has been prescribed, it has often been at a lower dose 50–100 (24/26–49/51) mg twice daily, and physicians report that SV users are often unable to reach the maximal target dose 200 (97/103) mg twice daily because of intolerance^4–8^. Real-world data show that the maximal target dose achievement rate for SV is only 27 to 38%^4,6^. There have been no studies (including the PARALLEL-HF trial conducted in Japan) examining the initiation of SV at a very low dose, such as 25 (12/13) mg twice daily in ambulatory patients, or which have compared this to the standard dose (SD) of 50 (24/26) mg twice daily^11^. In the present study, we examined the tolerability of SV commenced at a very low dose (VLD), and outcomes associated with this dose, compared to SD SV in patients with HFrEF.

## Methods

### Study population

This study was a single-center, prospective, observational study conducted at a tertiary university hospital^12,13^. The consecutive outpatients (≥ 18 years old) enrolled in this study from Jan 2017 to Sep 2018 had the following characteristics: (1) symptomatic chronic HF with New York Heart Association (NYHA) class II–IV, (2) left ventricular ejection fraction (LVEF) ≤ 40% in echocardiography, (3) use of a maximally tolerated dose of an ACEi/ARB for at least 4 weeks, and (4) use of other guideline-directed medical therapies (GDMT) for HF such as beta-blockers, mineralocorticoid receptor antagonists (MRA) or ivabradine, unless there were contraindications to these treatments^14^.

### Data collection

We reviewed medical records including laboratory data, medical history, pre-SV prescription therapy, and echocardiographic parameters at baseline. Patients were followed up every 1 to 6 months, and blood samples collected and physical examination, including measurement of office blood pressure (BP), performed at each visit. Echocardiographic parameters were assessed at least 12 weeks after SV treatment. Drug administration doses, including SV, beta-blocker, MRA and loop diuretics, were collected at the initial and follow-up visits. Only patients with a titration period of at least 6 months after starting SV were included. Composite clinical outcomes consisted of cardiovascular mortality and rehospitalization due to HF. This study was approved by the institutional review board of the Yonsei University Health System (2020-0401-001).

### Sacubitril/valsartan doses and titration patterns

Patients were stratified according to the dose of SV as follows; patients in the ‘VLD’ group received a dose of SV 25 mg twice daily and those in the ‘SD’ group received more than 50 mg or more twice daily, at the beginning of the study. VLD SV was prepared by the local pharmacy. The physician decision about which SV dose to start with was based on prior ACEi/ARB dose and BP. The pre-SV treatment dose of an ACEi/ARB were classified into two categories; a high-dose ACEi/ARB group defined as a total daily dose of enalapril > 10 mg or valsartan > 160 mg, or equivalent, and low-dose group, defined as a lower dose than the high-dose group. Patients on another ACEi/ARB had their dose adjusted to an equivalent dose of valsartan as done in the TITRATION trial^15^. Doses of beta-blockers were calculated as carvedilol equivalents^16^, and loop-diuretic dosing was calculated as furosemide equivalents^17^. Dose titration was considered in five categories; (1) maintenance, (2) dose-up, (3) dose-down, (4) dose-up & down and (5) discontinuation^15^.

### Tolerability according to adverse events and clinical outcomes

Tolerability was defined as the presence or absence of events including symptomatic hypotension (symptoms and systolic blood pressure, SBP < 100 mmHg at follow-up visit), worsening renal function (estimated glomerular filtration rate, eGFR < 30 mL/min/1.73 m^2^) and hyperkalemia (serum potassium > 5.5 mmol/L). The proportion of patients that achieved the maximal target dose without adverse events (or death) were assessed. The main clinical outcome was the composite of cardiovascular mortality or HF rehospitalization.

### Statistical analysis

Continuous quantitative variables were presented as mean ± standard deviation, and categorical data were presented as frequencies and percentages. We analyzed the differences between groups using the χ^2^ test or Fischer’s exact test for categorical variables and student’s t test or Wilcoxon rank sum for continuous variables. Time-to-event data for comparing the clinical outcomes between two groups were analyzed with the Kaplan–Meier curve and log-rank test. P-values < 0.05 represented statistically significant results. We performed all the analyses with SPSS version 25.0 statistical package (SPSS Inc., an IBM company, Chicago, IL, USA).

### Declaration of Helsinki

This study was approved by the Institutional Review Board of Severance Hospital (IRB no. 2020-0401-001) and conformed to the ethical guidelines from the 1975 Declaration of Helsinki. Informed consent was obtained from all subject and/or real guardian.

## Results

### Baseline characteristics

We analyzed 206 patients with HFrEF (mean age 63 ± 14 years, EF 26.4 ± 6.1%) that were treated with SV from January 2017 to Sep 2018 consecutively (median follow-up duration 285 days). Table 1 show the baseline clinical characteristics, including medical history, laboratory parameters and pre-SV treatment medications. Compared to patients in the SD group (n = 100), those commenced on the VLD (n = 106) of SV had a lower body mass index (BMI), lower SBP, lower LVEF and they were less likely to have a history of hypertension and diabetes mellitus. A higher proportion of patients in the VLD group had a SBP < 100 mmHg at the time of switching from an ACEi/ARB to SV.

**Table 1:** Baseline characteristics.

Variables	Very low-dose	Standard dose	p value
	(N = 106)	(N = 100)	
Male, n (%)	73 (68.9%)	72 (72.0%)	0.623
Age (years)	61.7 ± 15.2	63.5 ± 13.4	0.363
Body mass index (kg/m^2^)	23.5 ± 3.5	25.6 ± 4.1	< 0.001
**Etiology**			0.843
Ischemic cardiomyopathy, n (%)	31 (29.2%)	28 (28.0%)	
Non-ischemic cardiomyopathy, n (%)	75 (70.8%)	72 (72.0%)	
SBP (mm Hg)	102.8 ± 12.0	118.7 ± 13.8	< 0.001
SBP < 100 mmHg, n (%)	40 (37.7%)	6 (6.0%)	< 0.001
Heart rate (/min)	73.0 ± 14.6	71.6 ± 12.7	0.451
**NYHA classification, n (%)**			0.930
II	95 (89.6%)	90 (90.0%)	
III	11 (10.4%)	10 (10.0%)	
**Medical history, n (%)**			
Hypertension	55 (51.9%)	74 (74%)	0.001
Diabetes	23 (21.7%)	36 (36%)	0.022
Dyslipidemia	13 (12.3%)	19 (19.0%)	0.172
Myocardial infarction	20 (18.9%)	23 (23.0%)	0.443
Stroke	11 (10.4%)	9 (9.0%)	0.756
Atrial fibrillation	35 (33.0%)	32 (32.0%)	0.915
Chronic kidney disease	41 (38.7%)	37 (37.0%)	0.847
**Laboratory parameter**			
Blood urea nitrogen (mg/dL)	21.8 ± 10.6	22 ± 9.8	0.910
Creatinine (mg/dL)	1.1 ± 0.4	1.1 ± 0.4	0.854
eGFR (mL/min/1.73m^2^)	67.2 ± 19.9	66.2 ± 19.2	0.716
Sodium (mmol/L)	140.2 ± 2.6	140.9 ± 2.7	0.101
Potassium (mmol/L)	4.6 ± 0.5	4.6 ± 0.5	0.847
Hemoglobin (g/dL)	13.4 ± 1.8	13.8 ± 2.1	0.171
NT-proBNP (pg/mL)	2750.9 ± 4453.6	2311.1 ± 3662.1	0.475
**Echocardiograhic parameter**			
LVEF (%)	25.4 ± 6.1	27.3 ± 5.9	0.026
LVEDD (mm)	65.5 ± 9.4	64.7 ± 7.0	0.447
**Pre-SV treatment therapy**			
Angiotensin-converting enzyme inhibitor, n (%)	11 (10.4%)	13 (13.0%)	0.558
Angiotensin II receptor blocker, n (%)	95 (89.6%)	87 (87.0%)	0.558
Beta-blocker, n (%)	96 (90.6%)	93 (93.0%)	0.526
Mineralocorticoid receptor antagonist, n (%)	85 (80.2%)	75 (75.0%)	0.371
Loop diuretics, n (%)	89 (84%)	83 (83.0%)	0.852
Ivabradine, n (%)	21 (19.8%)	12 (12.0%)	0.127
Implantable cardioverter defibrillator, n (%)	40 (37.7%)	35 (35.0%)	0.723
Cardiac resynchronization therapy, n (%)	11 (10.4%)	15 (15.0%)	0.305

*SBP* systolic blood pressure, *NYHA* New York Heart Association, *LVEF* left ventricular ejection fraction, *eGFR* estimated glomerular filtration rate, *NT-proBNP* N-terminal prohormone of brain natriuretic peptide, *SV* sacubitril/valsartan.

### Dosing of sacubitril/valsartan

We analyzed baseline SBP and the pre-SV ACEi/ARB dose in relation to initial dose of SV (supplementary table 1). Patients with both a high SBP and a high ACEi/ARB dose were more likely to be prescribed SD SV (39% vs. 9.4%, p < 0.001) and patients with both a low SBP and a low ACEi/ARB dose were more likely to be commenced on VLD SV (34% vs. 5%, p < 0.001). Detailed descriptions about the medication types and dosing of pre-SV HF-GDMT are summarized in supplementary Table 2. In the SD group, equivalent valsartan and carvedilol doses were significantly higher compared to the VLD group.

### Change in physiological measures and clinical outcomes after initiation of sacubitril/valsartan

Table 2 shows changes in laboratory and echocardiographic parameters after initiating SV treatment. Follow-up SBP was lower and follow-up NT-proBNP level was higher in the VLD group when compared to the SD group, reflecting differences in these variables observed at baseline.

**Table 2:** Changes in laboratory and echocardiographic parameters after treatment with sacubitril/valsartan.

Variables	Very low-dose	Standard dose	p value
	(N = 106)	(N = 100)	
**SBP (mmHg)**			
Baseline	102.8 ± 12.0	118.7 ± 13.8	< 0.001
Follow-up	105.2 ± 16.0	115.3 ± 16.2	< 0.001
∆ Delta	2.4 ± 15.5	− 3.4 ± 17.8	0.014
**DBP (mmHg)**			
Baseline	65.2 ± 10.0	73.6 ± 12.2	< 0.001
Follow-up	64.9 ± 10.7	70.2 ± 13.1	0.002
∆ Delta	− 0.3 ± 11.1	− 3.4 ± 17.0	0.133
**Log NT-proBNP**			
Baseline	3.1 ± 0.6	3.0 ± 0.6	0.257
Follow-up	2.8 ± 0.7	2.5 ± 0.8	0.030
∆ Delta	− 0.3 ± 0.6	− 0.4 ± 0.6	0.096
**LVEDD (mm)**			
Baseline	65.5 ± 9.4	64.7 ± 7.0	0.447
FOLLOW-up	61.8 ± 9.7	59.8 ± 7.6	0.140
∆ Delta	− 3.8 ± 5.7	− 4.5 ± 6.3	0.420
**LVEF (%)**			
Baseline	25.4 ± 6.1	27.3 ± 5.9	0.026
Follow-up	34.2 ± 12.5	37.3 ± 11.2	0.081
∆ Delta	9.0 ± 12.3	9.9 ± 12	0.610

*SBP* systolic blood pressure, *DBP* diastolic blood pressure, *NT-proBNP* N-terminal prohormone of brain natriuretic peptide, *LVEDD* left ventricular end diastolic diameter, *LVEF* left ventricular ejection fraction.

**Figure 1:**
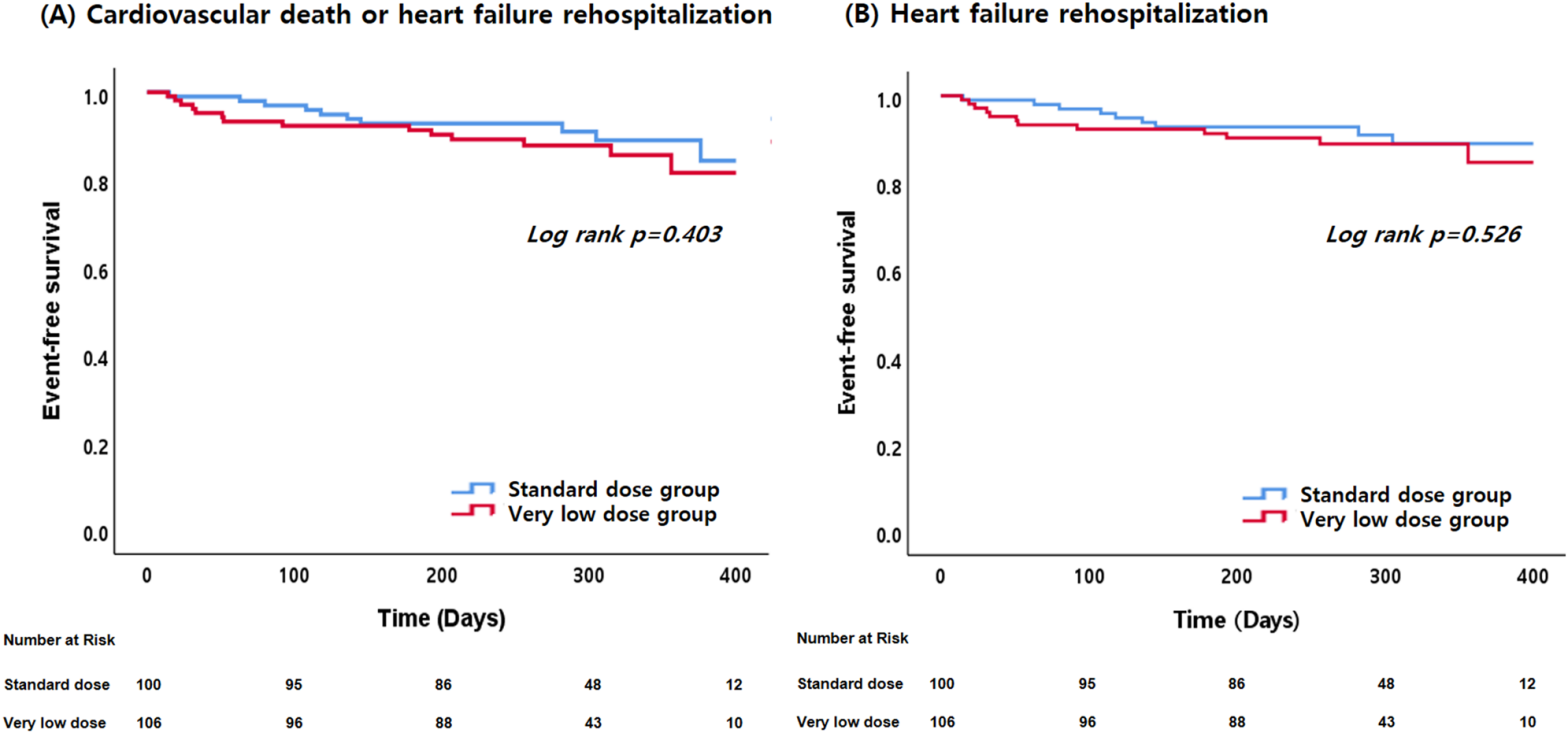
Kaplan–Meier curve for clinical outcomes between very low-dose sacubitril/valsartan and standard dose group. Event-free survival of the composite-end point (cardiovascular death or heart failure rehospitalization) (Panel **A**) and heart failure (HF) rehospitalization (Panel **B**).

In the overall population, SBP did not show a significant change from baseline to follow-up (from 111 ± 15 to 110 ± 17 mmHg, p = 0.751). However, after treatment with SV, SBP increased in the VLD group and decreased in the SD group, with a significant difference between the two groups (p = 0.014). In the overall population, NT-proBNP decreased following treatment with SV (from 2594 ± 4168 to 2199 ± 6253 pg/ml, p < 0.001), left ventricular end-diastolic dimension (LVEDD) decreased (from 65 ± 8 to 61 ± 9 mm, p < 0.001) and LVEF increased (from 26.4 ± 6.2 to 35.7 ± 12.0%, p < 0.001). The changes in NT-proBNP, LVEDD, and LVEF did not differ significant between the two dose-groups. Symptoms (NYHA classification) also improved after SV treatment (supplementary table 3). During the follow-up period, there were 27 composite clinical outcomes including 4 CV deaths and 23 HF rehospitalizations, with no significant difference between the two dose-groups (Fig. 1).

### Dose titration, the achievement of maximal target dose and adverse events

**Figure 2:**
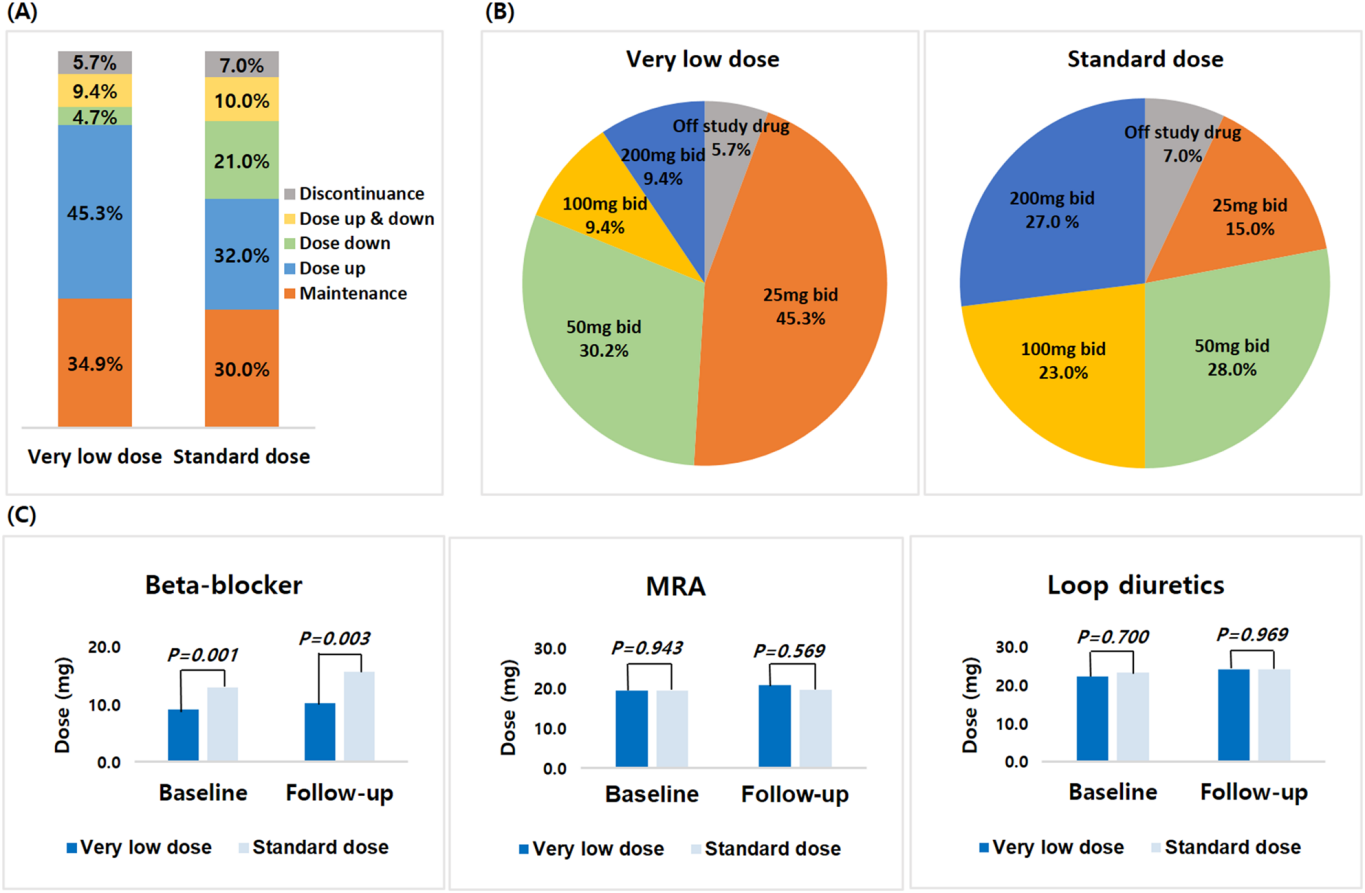
Heart failure guideline-directed medical therapies at follow-up compared with baseline for very low-dose and standard dose of sacubitril/valsartan groups. Panel (**A**) Pattern of dose titration, comparing very low-dose and standard dose sacubitril/valsartan groups (panel **A**). Pane (**B**) Proportions of patients achieving different sacubitril/valsartan dose levels in the very low-dose and standard dose sacubitril/valsartan groups (panel **B**). Panel C: Dosing of other heart failure treatments in the very low-dose and standard dose sacubitril/valsartan groups. The *p*-value represents a comparison between very low-dose and standard dose of sacubitril/valsartan at baseline and follow-up.

As shown in Fig. 2A, we found that the VLD group had more up-titrations (45.3% vs. 32.0%, p = 0.051), while patients in the SD group had more down-titrations (21.0% vs. 4.7%, p < 0.001). Discontinuation rates of SV were comparable between the two groups. Specifically, 6/106 patients (5.7%) in the VLD group and 7/100 patients (7.0%) in the SD group stopped SV for reasons other than death (P = 0.693). Table 3 shows the titration tolerability and adverse events during treatment with SV. Achievement of the maximal target dose of SV was higher in the SD group (Fig. 2B). The most common cause for intolerance during up-titration was dizziness, and more patients complained of dizziness in the SD group compared to the VLD group. The most common adverse event was hypotension, but there was no significant difference in the adverse events between the two dose-groups.

**Table 3:** The achievement rate of the maximal target dose of sacubitril/valsartan, tolerability, and adverse events.

	Very low dose	Standard dose	p value
	(N = 106)	(N = 100)	
Mean dose on last follow-up (mg)	115.0 ± 108.2	210.8 ± 137.6	0.001
Achieving maximal target dose (200 mg twice daily), n (%)	10 (9.4%)	27 (27.0%)	0.001
**Causes of titration intolerance, n (%)**			0.049
Dizziness	12 (11.3%)	24 (24.0%)	
Decreased SBP	7 (6.6%)	6 (6.0%)	
General weakness	0 (0%)	1 (1.0%)	
Decreased renal function	1 (0.9%)	2 (2.0%)	
Others	2 (1.9%)	5 (5.0%)	
**Adverse events, n (%)**			
Hypotension (SBP < 100 mmHg)	23 (21.7%)	16 (16.0%)	0.297
Symptomatic hypotension (SBP < 100 mmHg)	7 (7.0%)	3 (2.8%)	0.204
**Decreased renal function**			
(eGFR < 30 mL/min/1.73m^2^)	6 (5.7%)	3 (3.0%)	0.402
Hyperkalemia (K > 5.5 mg/dL)	6 (5.7%)	4 (4.0%)	0.752

*SBP* systolic blood pressure, *eGFR* estimated glomerular filtration rate.

We analyzed the prescription rate and equivalent dose of other GDMT medications for heart failure before and after treatment with SV (Fig. 2C, supplementary table 4). In the SD group, beta-blockers were used at higher doses at both baseline (12.9 ± 9.7, vs 9.0 ± 5.2 mg p = 0.001) and follow-up (15.6 ± 14.8 vs. 10.2 ± 7.9 mg, p = 0.003), compared to the VLD group. However, there were no significant differences in prescription rates and dose changes of beta-blockers during follow-up (or for MRA and loop diuretics).

## Discussion

In the present study, we demonstrated that patients with HFrEF initiated on a VLD of SV were characterized by lower SBP, BMI, and LVEF at baseline. Use of VLD SV was associated with an improvement in dyspnea, an increase in LVEF and a decrease in NT-proBNP and LVEDD, similar to that seen with the currently recommended SD of SV, and without any significant differences in adverse events, treatment discontinuation or clinical outcomes, when compared with SD of SV.

Based on the clinical benefits demonstrated in the PARADIGM-HF trial, both the ACC/AHA and ESC guidelines recommend SV treatment in patients with HFrEF^2,3^. However, according to a recent report about GDMT in HFrEF (CHAMP-HF, Change and Management of Patients with Heart Failure), 27% of patients did not receive an ACEi/ARB or SV despite absence of contraindications and only 13% of patients received SV^8^. Another analysis of the CHAMP-HF registry showed that only 10.8% patients took the target dose of ACEi/ARB, recommended by current guidelines^18^. These results suggest that initiation and up-titration of GDMT to target doses is often a clinical challenge in ordinary clinical practice.

The greatest challenge is in patients with a low BP (e.g. SBP < 100 mmHg) who are also those at highest risk of poor outcomes and who, potentially, have much to gain from effective therapies. A post-hoc analysis of the Systolic Heart Failure Treatment with the I_*f*_ Inhibitors Ivabradine (SHIFT) showed that the risk of all-cause mortality increased by 12% as baseline SBP decreased every 10 mmHg in chronic HFrEF patients^19^. The significance of low BP is further highlighted by the definition of “advanced heart failure” which includes patients with a SBP < 90 mmHg, and in whom treatments such as a left ventricular assist device or even heart transplantation may need to be considered^3,20^. In the real-world practice, it is easy to find patients with HFrEF with a low SBP, as shown in our study. Interestingly, in this study, the patients that physicians elected to start on VLD SV had other features of advanced heart failure. For example, they had a lower BMI which is known to identify higher-risk individuals, some of whom have cardiac cachexia, which is also part of the definition of advanced heart failure^21^. Finally, they had a lower LVEF (LVEF < 30%) and this too is also a recognized indicator of advanced HF^20^.

These high-risk HF patients have a poor tolerance of GDMT, and so it is inevitable that lower doses of guideline-recommended medications are prescribed in clinical practice (or these may not be prescribed at all). However, there have been few clinical studies that have examined the possibility of using lower than usual initial doses of GDMT in patients with HFrEF. We found that a VLD of SV was associated with similar improvements in symptoms, laboratory, echocardiographic parameters, and clinical outcomes, and had a similar adverse event profile in these vulnerable patients, compared to the SD group. Although our patients were not randomized, it is useful to compare our results with those from the PIONEER-HF trial in which hospitalized HFrEF patients with a systolic BP ≥ 100 mmHg were randomized to SV 50 mg bid (titrated, if possible, to 200 mg bid) or enalapril 2.5 mg bid (titrated to 10 mg bid)^22^. In PIONEER-HF, the lowest dose-level of SV (50 mg bid) led to a greater reduction in NT-proBNP than the equivalent randomized dose-level of enalapril (2.5 mg bid). Compared with enalapril, SV also reduced heart failure re-hospitalization, consistently, across all three dose-levels of study drug (50, 100 and 200 mg bid of SV versus 2.5, 5 and 10 mg bid of enalapril). While we cannot prove that those individuals who remained on VLD SV (i.e. 25 mg bid) obtained benefit from that dose, we can conclude from PIONEER-HF that the 49% who were successfully titrated to 50 mg bid or above likely did. It is also important to note that 94% of patients in the VLD group remained on SV. In the SD group, 78% of patients achieved a dose of SV of 50 mg bid or above and 93% remained on treatment. For comparison, 88% of patients in PIONEER-HF remained on SV of 50 mg bid or above at 12 weeks but 12% were off study drug (there was no 25 mg bid option in PIONEER-HF). Arguably, the availability of VLD SV resulted on more patients remaining on treatment in the present study and possibly some patients receiving treatment at all i.e. if it had not been for the availability of VLD SV, some patients might never have been started on treatment or would have been started on SD and had to discontinue treatment. Conversely, a forced-titration strategy, such as that used in randomized trials, might have led to achievement of higher doses that observed in our study, although other “real world” data are consistent with our experience, e.g. in the CHAMP-HF Registry, only 14% of patients received maximal target doses of SV^8^.

Our findings may be particularly relevant to Asian patients. In the PARADIGM-HF trial, the 1487 (18%) patients enrolled from the Asia–Pacific region had a lower BMI, lower SBP and lower prevalence of hypertension, findings comparable with our baseline data^23,24^. Clinical practice data from Taiwan demonstrated that only 15.8% patients could achieve the target SV dose (97/103 mg twice daily) after a 1-year titration pattern^25^. In addition, this study showed that very low doses of SV (12/13 to 24/26 mg daily) were prescribed. Therefore, future prospective studies to demonstrate the clinical role of a very low SV dose should be warranted, especially in the Asian population. However, it is important to reach the maximal target dose (200 mg twice daily) because the patients taking the full dose sacubitril/valsartan showed lower cardiovascular death or heart failure hospitalization then those taking lower than target doses of enalapril^26^. Following current guidelines, the clinician should strive to reach the maximal target dose as soon as possible.

Our study had some limitations. First, it had the inherent limitations of an observational study in a single center. Second, we used a very low dose of SV that is not generally available and had to be prepared locally by splitting a 50 mg SV tablet. Third, we analyzed a relatively small number of patients in a specific (Korean) population with a relatively short-term follow-up. A larger prospective, larger, longer term, randomized trial would be useful.

In conclusion, our study suggests that the initiation of VLD of SV (25 mg twice daily), in patients taking a low dose of an ACEi/ARBs, or with a low SBP, or both, is well tolerated and may be associated with similar outcomes an initial standard dose of 50 mg twice daily. This may be a useful clinical strategy in the many HFrEF patients who are currently denied SV and other therapies because of concerns about hypotension or who are unable to tolerate standard dose SV. Therefore, more attention should be warranted to our new strategy in a real practice, and individualized target dose for sacubitril/valsartan should be set based on SBP, body weight or severity of HF patients.

## Supplementary Information

Supplementary Information.
