# Supplementary material for: Clinical evidence of initiating a very low dose of sacubitril/valsartan: a prospective observational analysis
Source: Sci Rep. 2021 Aug 11;11:16335. doi: 10.1038/s41598-021-95787-w (PMC8358003; doi:10.1038/s41598-021-95787-w)
Supplement: Supplementary file 1 — Supplementary Information. [file 41598_2021_95787_MOESM1_ESM.docx]

**Supplementary table 1. Baseline systolic blood pressure and valsartan doses according to the doses at commencement**

|  | **Very low-dose** | **Standard dose** | **p value** |
| --- | --- | --- | --- |
| n (%) | **(N=106)** | **(N=100)** |  |
| SBP ≥ 100 mmHg, Valsartan ≥ 160 mg | 10 (9.4%) | 39 (39%) | <0.001 |
| SBP ≥ 100 mmHg, Valsartan < 160 mg | 56 (52.8%) | 55 (55%) |  |
| SBP < 100 mmHg, Valsartan ≥ 160 m | 4 (3.8%) | 1 (1%) |  |
| SBP < 100 mmHg, Valsartan < 160 mg | 36 (34%) | 5 (5%) |  |

SBP; systolic blood pressure

**Supplementary Table 2. Pre-sacubitril/valsartan medications and doses for heart failure**

|  | **Prevalence** | | |  | **Dose** | | **Equivalent dose** | |  |
| --- | --- | --- | --- | --- | --- | --- | --- | --- | --- |
|  | **Very low-dose (N=106)** | **Standard dose**  **(N=100)** | **p value** | | **Very low-dose**  **(N=106)** | **Standard dose**  **(N=100)** | **Very low-dose**  **(N=106)** | **Standard dose**  **(N=100)** | **p value** |
| **ACEi, n (%)** |  |  | 0.652 | |  |  | **Equivalent dose by Valsartan** | | |
| **Total daily dose** |  |  |  | |  |  | 114.6 ± 65.8 | 152.3 ± 95.8 | 0.281 |
| Captopril | 3 (2.8%) | 2 (2.0%) |  | | 65.6 ± 74.4 | 84.4 ± 92.8 | 113.3 ± 113.7 | 140.0 ± 141.4 | 0.816 |
| Perindopril | 4 (3.8%) | 5 (5.0%) |  | | 2.8 ± 1.5 | 3.6 ± 0.9 | 110.0 ± 60.0 | 144.0 ± 35.8 | 0.323 |
| Ramipril | 3 (2.8%) | 4 (4.0%) |  | | 3.3 ± 1.4 | 6.3 ± 4.3 | 106.7 ± 46.2 | 200.0 ± 138.6 | 0.280 |
| Others | 1 (0.9%) | 2 (2.0%) |  | |  |  |  |  |  |
| **ARB, n (%)** |  |  | 0.001 | |  |  |  |  |  |
| **Total daily dose** |  |  |  | |  |  | 70.3 ± 35.2 | 112.6 ± 76.8 | <0.001 |
| Candesartan | 14 (13.2%) | 16 (16.0%) |  | | 6.6 ± 3.4 | 10.8 ± 5.0 | 65.7 ± 33.7 | 107.5 ± 50.0 | 0.012 |
| Losartan | 31 (29.2%) | 8 (8.0%) |  | | 26.2 ± 6.7 | 46.9 ± 33.9 | 83.9 ± 21.6 | 150.0 ± 108.5 | 0.129 |
| Valsartan | 49 (46.2%) | 53 (53.0%) |  | | 61.2 ± 38.2 | 92.1 ± 63.5 | 61.2 ± 38.2 | 92.1 ± 63.5 | 0.004 |
| Telmisartan | 0 (0%) | 6 (6.0%) |  | |  | 53.3 ± 20.7 |  | 213.3 ± 82.6 |  |
| Others | 1 (0.9%) | 4 (4.0%) |  | |  |  |  |  |  |
| **Beta-blocker, n (%)** |  |  | <0.001 | |  |  | **Equivalent dose by Carvedilol** | | |
| **Total mean dose** |  |  |  | |  |  | 9.0 ± 5.2 | 12.9 ± 9.7 | 0.001 |
| Bisoprolol | 45 (42.5%) | 17 (17.0%) |  | | 1.8 ± 0.9 | 2.7 ± 1.7 | 9.0 ± 4.4 | 13.4 ± 8.3 | 0.047 |
| Carvedilol | 47 (44.3%) | 71 (71.0%) |  | | 8.2 ± 5.1 | 12.2 ± 10 | 8.2 ± 5.1 | 12.2 ± 10.0 | 0.002 |
| Nebivolol | 4 (3.8%) | 5 (5.0%) |  | | 3.4 ± 1.9 | 4.0 ± 1.4 | 17.2 ± 9.4 | 20.0 ± 6.8 | 0.618 |
| **MRA** | 85 (80.2%) | 75 (75.0%) | 0.371 | | 19.3 ± 9.0 | 19.2 ± 8.3 |  |  | 0.943 |

ACEi; angiotensin-converting enzyme inhibitor, ARB; angiotensin II receptor blocker, MRA; mineralocorticoid receptor antagonist

**Supplementary Table 3. Changes in other laboratory and clinical parameters after treatment with sacubitril/valsartan**

| **Variables** | **Very low-dose** | | **Standard dose** | | **p value** | |  |
| --- | --- | --- | --- | --- | --- | --- | --- |
|  | **(N=106)** | | **(N=100)** | |  |  |  |
| Follow-up NT-proBNP (pg/dL) | | 2469.7 ± 6484.2 | | 2056.1 ± 6467.1 | | 0.670 | |
| ∆ Delta NT-proBNP (pg/dL) | | -504.9 ± 6873.5 | | -278.1 ± 4731.3 | | 0.804 | |
| Follow-up BUN (mg/dL) | 22.3 ± 12.3 | | 21.4 ± 10.3 | | 0.574 | |  |
| ∆ Delta BUN (mg/dL) | 0.5 ± 9.3 | | -0.6 ± 7.2 | | 0.357 | |  |
| Follow-up Creatinine (mg/dL) | 1.2 ± 0.6 | | 1.2 ± 0.6 | | 0.797 | |  |
| ∆ Delta creatinine (mg/dL) | 0.1 ± 0.4 | | 0.1 ± 0.5 | | 0.666 | |  |
| Follow-up eGFR (mL/min/1.73m^2^) | 63.8 ± 21.6 | | 64.3 ± 21.6 | | 0.858 | |  |
| ∆ Delta eGFR (mL/min/1.73m^2^) | -3.5 ± 10.5 | | -2.3 ± 10.5 | | 0.419 | |  |
| Follow-up Sodium (mmol/L) | 140.8 ± 2.7 | | 141 ± 3.3 | | 0.776 | |  |
| ∆ Delta Sodium (mmol/L) | 0.6 ± 3.0 | | 0.3 ± 3.0 | | 0.435 | |  |
| Follow-up Potassium (mmol/L) | 4.8 ± 0.5 | | 4.7 ± 0.5 | | 0.467 | |  |
| ∆ Delta Potassium (mmol/L) | 0.2 ± 0.7 | | 0.1 ± 0.6 | | 0.449 | |  |
| Follow-up NYHA class, n (%) |  | |  | | 0.858 | |  |
| I | 35 (33.0%) | | 34 (34.0%) | |  | |  |
| II | 67 (63.2%) | | 64 (64.0%) | |  | |  |
| III | 4 (3.8%) | | 2 (2.0%) | |  | |  |

NT-proBNP; N-terminal prohormone of brain natriuretic peptide, BUN; blood urea nitrogen, eGFR; estimated glomerular filtration rate, NYHA; New York Heart Association

**Supplementary Table 4. Changes in the usage and dosing of medications during follow-up**

|  | **Very low-dose (N=106)** | **Standard dose**  **(N=100)** | **p value** | **Very low-dose (N=106)** | **Standard dose**  **(N=100)** | **p value** |
| --- | --- | --- | --- | --- | --- | --- |
| **Beta-blocker, n (%)** |  |  |  | Equivalent dose by carvedilol | |  |
| Baseline | 96 (90.6%) | 93 (93.0%) | 0.526 | 9.0 ± 5.2 | 12.9 ± 9.7 | 0.001 |
| Last follow-up | 96 (90.6%) | 83 (83.0%) | 0.108 | 10.2 ± 7.9 | 15.6 ± 14.8 | 0.003 |
| ∆ Delta of dose |  |  |  | 1.7 ± 6.6 | 2.8 ± 12.5 | 0.480 |
| **MRA, n (%)** |  |  |  |  |  |  |
| Baseline | 85 (80.2%) | 75 (75.0%) | 0.371 | 19.3 ± 9.0 | 19.2 ± 8.3 | 0.943 |
| Last follow-up | 78 (73.6%) | 65 (65.0%) | 0.181 | 20.6 ± 12.9 | 19.4 ± 11.3 | 0.569 |
| ∆ Delta of dose |  |  |  | 2.0 ± 11.0 | 1.3 ± 12.8 | 0.745 |
| **Loop diuretics, n (%)** |  |  |  | Equivalent dose by furosemide | |  |
| Baseline | 89 (84.0%) | 83 (83.0%) | 0.852 | 22.2 ± 16.2 | 23.1 ± 13.4 | 0.700 |
| Last follow-up | 82 (77.4%) | 74 (74.0%) | 0.574 | 24.1 ± 23.5 | 24 ± 18.1 | 0.969 |
| ∆ Delta of dose |  |  |  | 3.9 ± 23.4 | 3.7 ± 20.4 | 0.939 |

MRA; mineralocorticoid receptor antagonist
